# Adaptation and phenotypic diversification of *Bacillus thuringiensis* biofilm are accompanied by fuzzy spreader morphotypes

**DOI:** 10.1038/s41522-022-00292-1

**Published:** 2022-04-13

**Authors:** Yicen Lin, Xinming Xu, Gergely Maróti, Mikael Lenz Strube, Ákos T. Kovács

**Affiliations:** 1 Bacterial Interactions and Evolution Group, DTU Bioengineering, Technical University of Denmark, 2800 Lyngby, Denmark; 2 Institute of Plant Biology, Biological Research Center, ELKH, 6726 Szeged, Hungary; 3 Bacterial Ecophysiology and Biotechnology Group, DTU Bioengineering, Technical University of Denmark, 2800 Lyngby, Denmark

**Keywords:** Biofilms, Molecular evolution

## Abstract

*Bacillus cereus* group (*Bacillus cereus sensu lato*) has a diverse ecology, including various species that produce biofilms on abiotic and biotic surfaces. While genetic and morphological diversification enables the adaptation of multicellular communities, this area remains largely unknown in the *Bacillus cereus* group. In this work, we dissected the experimental evolution of *Bacillus thuringiensis* 407 Cry- during continuous recolonization of plastic beads. We observed the evolution of a distinct colony morphotype that we named fuzzy spreader (FS) variant. Most multicellular traits of the FS variant displayed higher competitive ability versus the ancestral strain, suggesting an important role for diversification in the adaptation of *B. thuringiensis* to the biofilm lifestyle. Further genetic characterization of FS variant revealed the disruption of a guanylyltransferase gene by an insertion sequence (IS) element, which could be similarly observed in the genome of a natural isolate. The evolved FS and the deletion mutant in the guanylyltransferase gene (Bt407Δ*rfbM*) displayed similarly altered aggregation and hydrophobicity compared to the ancestor strain, suggesting that the adaptation process highly depends on the physical adhesive forces.

## Introduction

Multicellularity, the assemblage of differentiated cells, has recently gained attention as an evolutionary strategy among microbes^1^. Although a well-accepted concept for eukaryotes, it took several decades to generally acknowledge that nearly all bacteria are capable of multicellular behaviors^2^. Yet, bacteria are nowadays broadly harnessed to study the evolution of multicellular traits due to their simpler characteristics. Among general classes of multicellular bacteria, aggregation is regarded as one of the most critical classes. Embedded within the extracellular matrix (ECM) of biofilms, aggregation-mediated cell-cell adhesion provides a fitness advantage for bacteria in various ways^3^. For instance, by holding cells together, ECM prevents the noxious influence of external toxic substances like antimicrobial compounds and enables community members to share enzymes and retain liquid repellency^4,5^. In *Myxococcus xanthus*, ECM is responsible for coordinated movement such as swarming motility by building cell collectives and triggering pilus retraction^6^. The relatively large size of multicellular aggregates might also confer a selective advantage compared with individual cells when facing predation^7^. More importantly, in environments where resources are inadequate for unicellular growth, aggregation can support direct access to nutrients produced by neighboring cells^8–10^. In analogy to nutrient transport in the veins of the animal body, secreted compounds can be dispersed through channels within structured biofilms. Likewise, multicellular aggregates also have a predominant fitness advantage over solitary cells on plant leaves^11,12^. The advantages of multicellular traits are plentiful, primarily due to the larger microbial biomass created through physical adhesion. These benefits, primarily due to improved resource uptake among cooperative microbes, can offset the negative effects that multicellular-like behavior brings, such as impaired motility and increased competition for food resources with their unicellular predecessors owing to higher cell density^3,13,14^.

Experimental evolution studies have been utilized to explore bacterial adaptive diversification for decades. One of the simplest models uses glass tubes culturing *Pseudomonas fluorescens* under static condition^15^. Driven by spatial heterogeneity and competition for vacant niches, *P. fluorescens* rapidly diversifies into three distinct colony morphotypes. The wrinkled-spreader morphotype with multicellular characteristics forms a self-supporting mat at the air-liquid surface. Owing to its simplicity and reproducibility, this static microcosm serves as a model to study evolutionary diversification that eventually expands our ecological and genetic understanding of *P. fluorescens*^16–19^. Additional simple setups have also been successfully applied to study evolution outcomes, such as colonies on solid agar plates^20,21^, submerged biofilms in microtiter plates^22^, and in silico models simulating static systems^23,24^. Despite the variations among these experimental setups, the generally applied spatially structured environments provide ecological opportunities in the form of distinct niches, where the diversifying selection is driven by resource competition. When such spatial structure is destroyed by shaking, the diversification is also eliminated^15^. However, when a vacant niche is constructed, even in shaking conditions, heterogeneity within biofilms can still provide ecological opportunities for adaptive diversification, as demonstrated in a bead model^25^. While the evolution of biofilms has been mostly studied in Gram-negative bacteria, including *Pseudomonas spp*. and *Burkholderia cenocepacia*, much less attention has been given to Gram-positive bacteria. Within Gram-positive and spore-forming bacteria, *Bacillus subtilis* has been exploited to reveal that adaptive specialization readily occurs under aerated cultivation via mutations that influence the regulation of biofilm development^26^. Focusing on the air-liquid floating biofilm, called pellicle that creates a highly structured environment, pellicles of *B. subtilis* underwent significant evolutionary diversification after ca. 200 generations, including an exploitative interaction among different evolved morphotypes^27^. Similarly, reduced spatial heterogeneity or hampered motility can also select for higher matrix production^28^. Recent works started to exploit *Bacilli* to understand how the colonization of a plant host influence bacterial evolution^29–31^.

*Bacillus cereus* group species are widely observed in soil samples. A consensus view is that *B. cereus sensu lato* can proliferate either within animal hosts or in the rhizosphere, exhibiting either pathogenic or symbiotic lifestyles^32^. Switching to multicellular-like behavior is one of the key strategies to thrive in these diverse niches. For example, in the guts of arthropods, *B. cereus* can grow by creating multicellular structures^33,34^. Another example demonstrated how *B. cereus* grew in soil-extracted organic matter medium by employing a growth of multicellular mode embedded in extracellular matrix^35^. Collectively, these examples indicate a conserved capacity among *B. cereus* species to grow in multicellular structures like cell aggregates and chains.

Despite the considerable amount of literature on the multicellular growth of *B. cereus* species, there has been little evidence of experimental evolution and ecological benefits of multicellular structures in these bacteria. In this study, we seek to explore how *Bacillus thuringiensis* (*Bt*) adapts to cycles of biofilm formation and illustrate that *Bt* could adopt a multicellular lifestyle to retain fitness advantage in a bead colonization model. This model routinely selects for cells colonizing the surface of plastic beads and subsequently dispersing from them and therefore it creates a simplified selection for the bacterial life cycle.

## Results

### The evolution of biofilm on plastic beads is accompanied by the diversification of Bt407 into a distinct morphotype

**Fig. 1:**
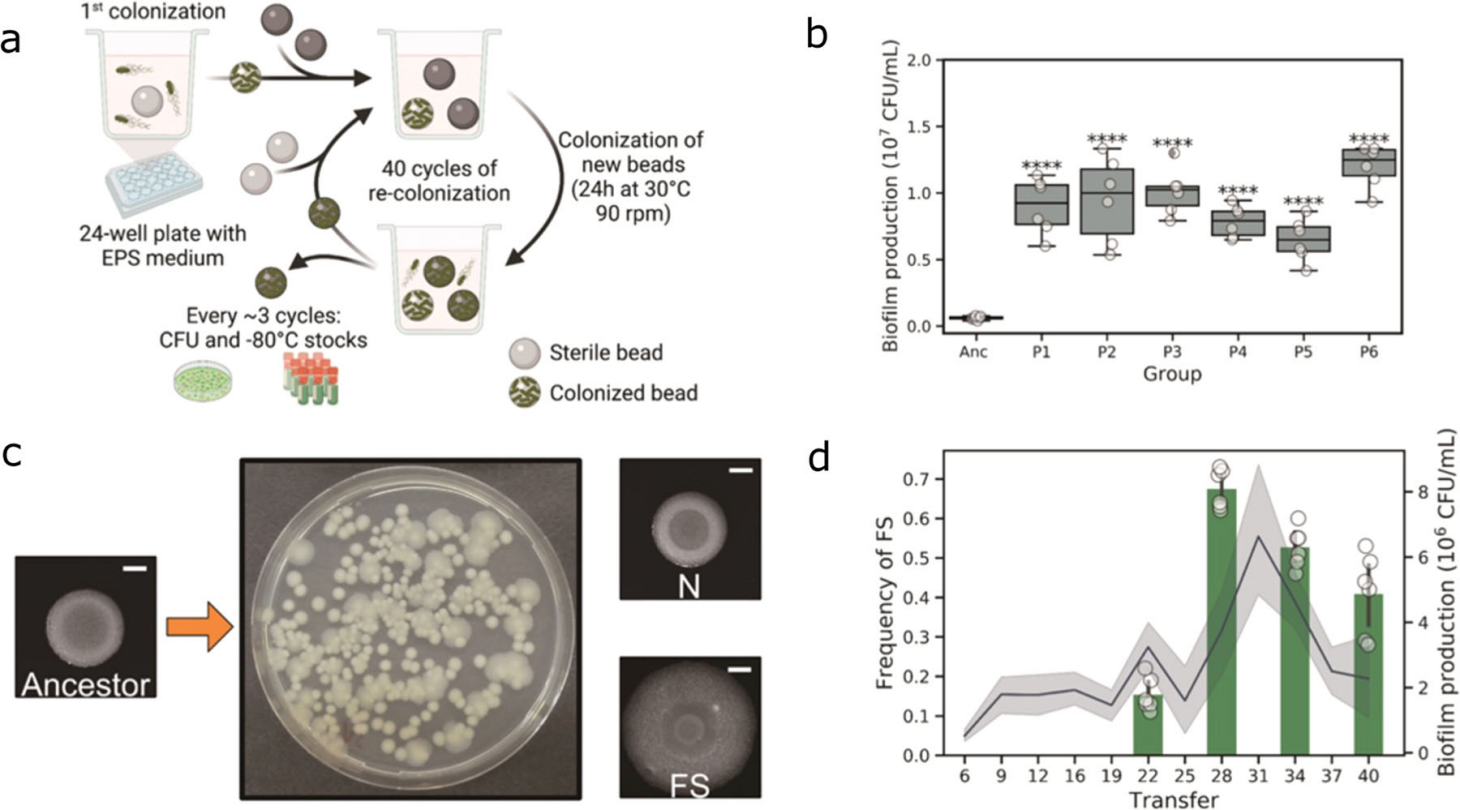
Evolution of nylon bead-associated biofilms of Bt407 diversified into a distinct morphotype. **a** Schematic representation of the bead-based experimental evolution setup. **b** Enhanced biofilm productions were revealed by CFU analysis of all final evolved populations compared with the ancestor (*n* = 6 biologically independent samples). Boxes indicate Q1–Q3, lines indicate the median, and bars span from max to min. Asterisks indicate significant differences between each group and the ancestor (****p < 0.0001; One-way ANOVA followed by Dunnett’s multiple comparison tests). **c** Distinct colony morphotypes of the evolved variants were evidenced on the LB agar medium. N and FS indicate normal and fuzzy spreader morphotypes, respectively. Scale bars indicate 5 mm. **d** Bar plot represents the relative frequencies of the FS morphotype (*n* = 6). Line plot represents the average biofilm productivity along with the experimental evolution. Error bars and shaded area indicate the standard error of the mean in the bar chart and line chart, respectively.

To test the adaptive diversification of *B. thuringiensis* 407 Cry- (Bt407), six independent populations were experimentally evolved as biofilms. We utilized the nylon bead-based biofilm experimental evolution system developed by Poltak and Cooper^25^, but included three beads in each new inoculation step: one colonized bead from the previous step and two sterile beads that were subsequently used for the next transfer or to determine the bacterial cell counts (Fig. 1a). Around every three transfers, biofilm developed on one of the beads was dispersed using sonication, and cells were plated on lysogeny broth (LB) agar plates for colony-forming unit (CFU) counting. Biofilm productivity (i.e., cell counts of the biofilms developed on the beads) increased gradually compared with the initial inoculum until transfer 31, after which a decrease was observed (Supplementary Fig. 1). In the end, all six biofilm populations displayed significantly enhanced biofilm production, suggesting the bead biofilm model serves as a great tool to select for biofilm-forming lineages (Fig. 1b). On the contrary, six experimentally evolved populations that were continuously cultivated under planktonic conditions did not exhibit a significant difference in their biofilm formation ability compared with the ancestor (Supplementary Fig. 2). Notably, the number of generations might differ in the two experimental systems.

In structured environments, microbes can diversify into phenotypically different variants, which are identified as morphotypes as they exhibit distinguishable colony morphologies. Biofilms on the beads are regarded as a classical example of such a structured environment. In all evolved biofilm populations, a distinct morphotype, demonstrating large and surface colonizing colony with relatively translucent edges (fuzzy spreader), was identified on LB agar plates (Fig. 1c). On the contrary, no such evolved variants were observed in planktonic evolved populations. The other colony morphotype was indistinguishable from the ancestor and therefore was termed as normal (N). To unravel the evolutionary history of phenotypic diversification and determine the frequencies of FS in all populations, fractions of frozen stocks from selected evolutionary time points were serially diluted in 0.9% sodium chloride solutions and plated onto LB agar. FS morphotypes were first detected around transfer 22 with a maximum frequency at transfer 28. Notably, the increased frequencies of FS coincided with the maximum biofilm productivity, implying FS morphotype may act as a biofilm-specialist in the bacterial populations (Fig. 1d).

### The phenotypic variation affects preference of biofilm formation in Bt407 evolved isolates

**Fig. 2:**
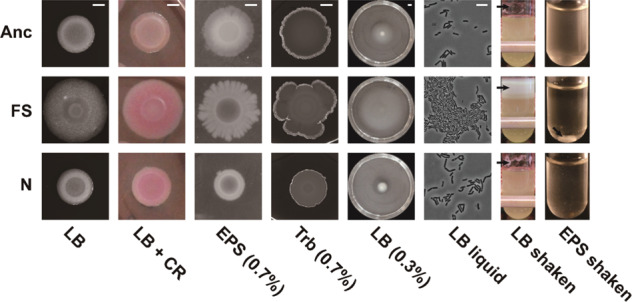
Phenotypic determination of the evolved variants. From left to right: colony morphologies, Congo red uptake, swarming motility on EPS and Trb medium, swimming motility, cellular morphologies in LB liquid, biofilm formation in LB and EPS medium in shaken cultures. Anc, FS, and N indicate the ancestor, FS, and N morph variants, respectively. Scale bars in colony and surface motility images indicate 5 mm and that in single cellular morphologies indicates 10 µm across all groups. Arrows indicate the formed biofilm in the LB shaken samples.

To further elucidate the phenotypic variations among morphotypes, we compared fundamental cellular differentiation properties of the derived two morphotypes from population 1 (P1) testing biofilm formation ability and motility (Fig. 2 and Supplementary Fig. 3). The FS morphotype displayed increased Congo red uptake (Supplementary Table 1), suggesting that the FS variant had enhanced extracellular matrix production such as exopolysaccharides compared with the ancestor. In addition to a slightly increased wrinkleality, the FS colonies demonstrated enhanced spreading motility on both EPS (29 mm ± 4 mm) and Trb media plates (33 mm ± 6 mm) with 0.7% of agar. On EPS agar, normal variants (11 mm ± 3 mm) and the ancestor (15 mm ± 4 mm) demonstrated similar surface motility on Trb agar; no significant difference was found between normal variants (15 mm ± 5 mm) and the ancestor (14 mm ± 4 mm) either. The swimming motility assays revealed that, while FS variants showed dramatically higher swimming motility (73 mm ± 8 mm), the *N* variants were unable to swim. The swimming ability of the ancestor was between the two evolved isolates (22 mm ± 7 mm). Intriguingly, FS variants displayed dendritic (branched) spreading, especially on EPS medium. Dendritic formation of colonies depends on nutrient resources and is promoted under lower nutrient conditions. The dendritic colony of the FS variant might represent an alternative surface translocation strategy possibly contributing to fitness during the selection process. While the N morphotype showed comparable colony morphology to the ancestor on LB agar medium, it displayed increased uptake of Congo red and decreased colony spreading ability on both spreading plates, suggesting a distinct influence on biofilm development compared to the ancestor. However, the statistical analysis indicated no significant difference was found between the N variant and the ancestor (Supplementary Table 1). These properties of FS and N morphotypes from population 1 could also be demonstrated for isolates from the other five populations (Supplementary Fig. 3), highlighting parallel evolution in all representative populations.

Motivated by the generally positive correlation between Congo red binding and biofilm formation^36,37^, biofilm formation of the morphotypes was tested in shaken LB cultures. Both evolved variants exhibited increased biofilm formation on the tube walls, especially the FS variant. In the nutrition limited EPS medium, the FS variant largely formed non-attached aggregates in the liquid fraction (30.91 mm^2^ ± 13.74 mm^2^ in area quantification by ImageJ), further suggesting that the selection led to multicellular-like behavior by the FS morphotype. The *N* variant showed intermediate biofilm-related phenotypes. Besides, the cells of FS morphotype exhibited dense and aggregated behavior as observed under the microscope, again suggesting an elevated biofilm formation ability. Taken together, the FS variant exhibited a dramatically higher degree of multicellular-like behavior compared with the ancestor, while the N morphotypes might serve as a general member of the microbial population contributing to a different function within the biofilm.

### Evolution of synergistic association of evolved isolates in bead biofilms

**Fig. 3:**
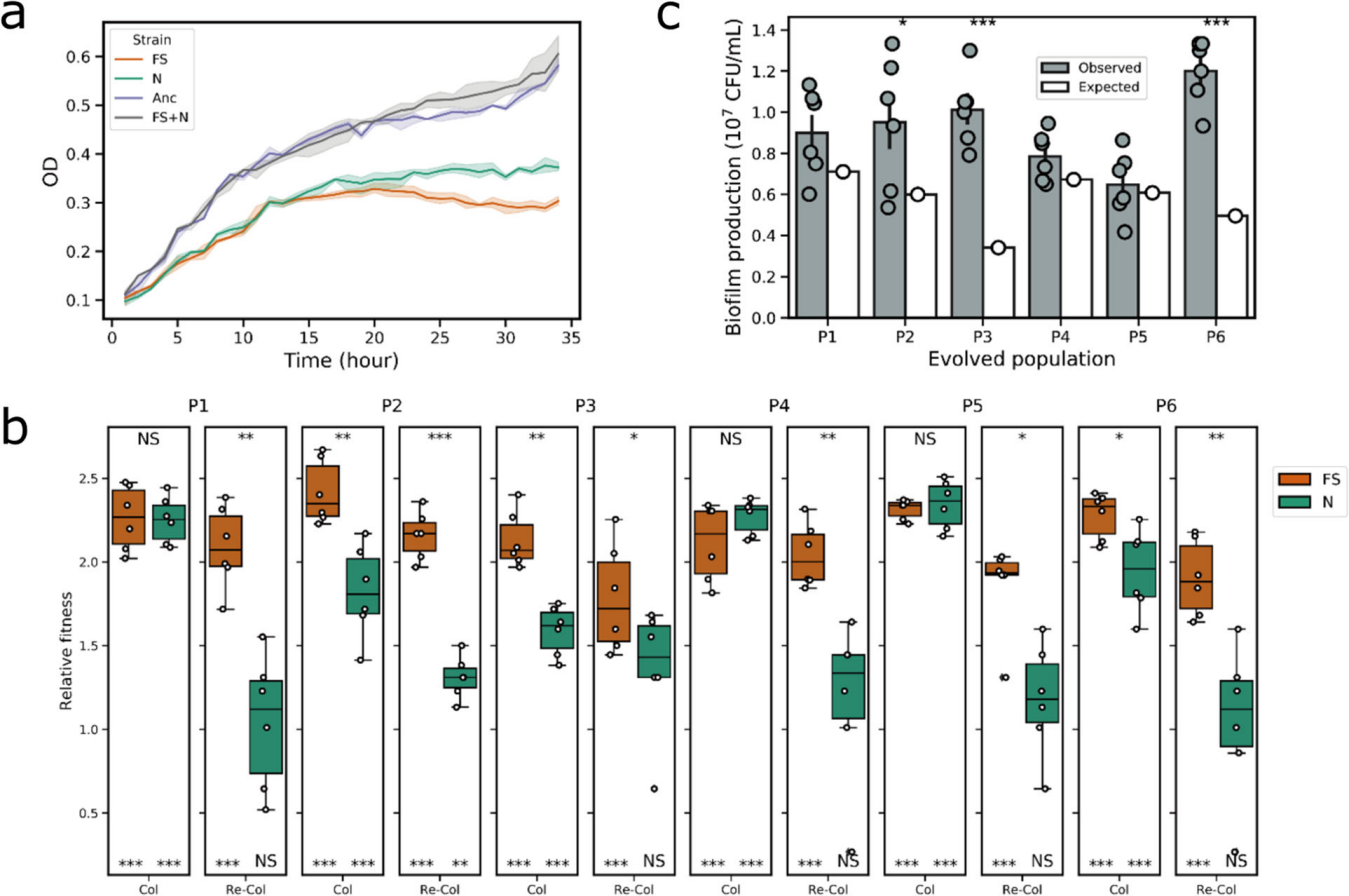
Morphotype fitness in planktonic and biofilm-forming conditions. **a** Growth properties of the evolved variants and the ancestor (*n* = 6) in EPS medium at 30 °C with continuous shaking (90 rpm). **b** Relative fitness of the evolved variants in the bead biofilms compared with the ancestor. Columns indicate colonization and re-colonization of evolved populations, respectively (P1-P6). The orange color indicates FS variants and the green color indicates *N* variants. Boxes indicate Q1–Q3, lines indicate the median, and bars span from max to min. Asterisks at the top indicate significant differences between FS and N morph variants. Asterisks at the bottom indicate significant differences between each group and the ancestor (*p < 0.05, **p < 0.01, ***p < 0.001; One-way ANOVA followed by Dunnett’s multiple comparisons tests). **c** Observed and expected biofilm productions of six evolved populations (P1–P6). Expected productivities were calculated as the product of the proportion of the evolved variant in each population and their productivities in monocultures. For observed biofilm productions, error bars indicate the standard error of the mean of independent biological samples (*n* = 6). Asterisks indicate significant differences between the observed and expected biofilm productions (*p < 0.05, ***p < 0.001; two-tailed *t*-test with Welch’s corrections).

Bacterial evolution often involves a trade-off between planktonic growth and biofilm formation. Importantly, the theory that trade-off in competition-colonization facilitates adaptive radiation is widely accepted. To reveal any trade-off in the evolved morphotypes, the variants were quantitatively tested in planktonic and biofilm states and compared to the ancestor strain. Both morphotypes exhibited reduced growth in planktonic cultures compared with the ancestor, suggesting that higher fitness in biofilm formation is at the expense of planktonic doubling time (Fig. 3a). Nonetheless, when the two morphotypes were incubated together, the growth rate of the well-mixed liquid cultures was restored to the level of the ancestor (Fig. 3a), which might point towards metabolic or other growth-promoting interaction between the two evolved variants.

Relative fitness of representative morphotypes from all evolved populations was calculated as the ratio of Malthusian parameter described by Lenski et al.^38^. Testing the evolved variants from all six populations confirmed increased fitness compared to the ancestor when cultures colonized the nylon beads. All FS variants exhibited stable and robust fitness advantage over the ancestor, relative fitness ranging from 2.11 to 2.41 on average (Fig. 3b), while the *N* variants also had increased fitness, while displaying higher variability, 1.58 to 2.34 on average. When two-cycle bead colonization was tested, termed re-colonization, the fitness advantage of FS variants remained high compared to the ancestor (1.78–2.15). On the contrary, the relative fitness of the N morphotype during the bead re-colonization was comparable to the ancestor. The statistical comparison between FS and *N* variants further confirmed the latter ones had reduced re-colonization fitness. Notably, while the FS and N isolates from three populations (P2, P3, and P6) showed significantly different fitness during colonization, isolates from the other three populations exhibited comparable fitness, suggesting that the *N* variants across evolved populations may not have acquired similar fitness advantage and phenotypic adaptation, unlike FS variants. Noteworthy, re-colonizing ancestor cells produced dramatically less biofilms than colonizing cells (*p* < 0.01, Supplementary Fig. 4), indicating a strong selection bottleneck for immigrating cells from old biofilms to new ones.

Finally, using the determined biofilm yield of monocultures, the expected productivities in the final mix cultures were calculated based on their frequencies in the mix^25^. Not surprisingly, the observed yield of the mix cultures was higher than the expected productivities (Fig. 3c), which was in accordance with the previous literature^25,27,29^. Notably, the difference between the predicted and observed productivity was higher in populations P2, P3, and P6, in which the FS and *N* variants exhibited significantly different colonization fitness values.

### Multicellular characteristics of evolved FS morphotypes confer ecological benefits

**Fig. 4:**
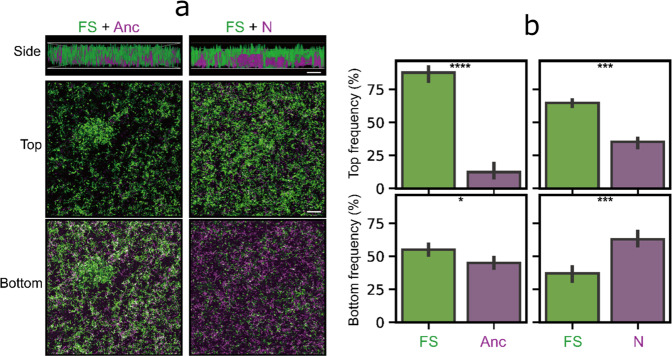
Biofilm architectures of the competitive experiments between the FS morphotype and the other two variants. **a** Images represent the aerial view of mixed biofilms of the GFP-labeled FS morphotype and the mKate-labeled *N* variant or ancestor. Scale bars indicate 50 µm. The image is representative of three biological replicates. **b** Frequencies of each strain in the bead biofilms analyzed by ImageJ. The bars and the errors represent the mean (*n* = 3) and the standard error of the mean, respectively. Asterisks indicate significant differences (*p < 0.05, ***p < 0.001, ****p < 0.0001; two tailed *t*-test with Welch’s corrections).

The spatial organization of evolved variants within biofilms influences the relative fitness of the population and affects competitive or cooperative traits^25,39–41^. Therefore, to further explore the microbial populations, variants of population P1 were fluorescently labeled along with the ancestor, and the spatial distribution of each morphotype and ancestor were imaged. Confocal laser scanning microscopy (CLSM) imaging and frequency analysis demonstrated that FS variants gained a substantial competitive advantage over the ancestor, exhibiting large multicellular clusters in the biofilms (Fig. 4). While the FS variant strongly outcompeted the ancestor, when co-cultured with the N variant, the FS morphotype produced smaller aggregates compared with the FS-ancestor co-cultures. Notably, the normal variants occupied the bottom layer of the biofilms, while the FS morphotype resided in the upper region, suggesting a spatial arrangement among the evolved variants (Fig. 4a). Following statistical analysis revealed FS and *N* variants had significant advantages against each other at the top and bottom layer of the biofilm structure, respectively (Fig. 4b). The spatial diversification of the morphotypes might explain the increased total biofilm yield compared with the expected calculation. Furthermore, the quantification of CFU within co-cultured biofilms also confirmed the competitive advantage of FS variants (Supplementary Fig. 4B).

The structural characteristics of the mixed biofilms might confer the top layer-residing FS variants significant benefits in terms of dispersal and reattachment. While only a few studies have experimentally examined biofilm dispersal of Gram-positive *Bacilli*^42^, an adapted methodology was applied to monitor the dispersal of three strains into the new environment^43–45^. Briefly, mature biofilms were incubated with a fresh medium, then dispersal of bacteria was quantitatively monitored over time. As expected, significantly more dispersed FS cells were present in the medium compared with the dispersal of the other two strains (Supplementary Fig. 5).

### Genomic characterization of evolved isolates

To dissect the genetic alterations of the evolved morphotypes, genomic DNA of selected FS and N morphotypes from each population at the end of the experiment were extracted and the genome sequences were determined by the combination of Illumina and Nanopore sequencing (Supplementary Dataset 1). Surprisingly, the genome comparison revealed 1–7 mutations in the evolved isolates compared to the assembled genome of the ancestor. While some of the mutations found in most isolates (e.g., two mutations were identified in six isolates from three populations), no SNP was found to be present in all isolates, highlighting the variance of the evolutionary outcome. Nevertheless, no common SNP was identified in the FS variants compared to the N morphotypes. Therefore, we took advantage of the genome assemblies provided by the hybrid assembly based on the Illumina and Nanopore reads. This analysis revealed numerous genome rearrangements in the evolved morphotypes (Supplementary Dataset 2).

Different mutations in *spo0F*, a gene coding sporulation initiation phosphotransferase, were detected in five evolved populations, suggesting parallel losses or alterations of the sporulation life stage during adaption to the constantly changing environment. When cultivated in an excess of nutrients, many *Bacillus* species can lose their sporulation capacity according to different laboratory evolution experiments^46–48^. Likewise, our previous evolution experiment showed a decreased sporulation efficiency in evolved isolates compared with Bt407^31^. Notably, compared to the ancestor and the *N* variants, all six FS isolates contained an insertion element in the BTB_c54660 gene, annotated as cupin domain-containing protein. Subsequent detailed analysis revealed that this gene encodes a mannose-1-phosphate guanylyltransferase (GDP) enzyme, which belongs to the glycosyltransferase family A. In numerous organisms, GDP involves in GDP-mannose biosynthesis, which acts as the precursor for mannose residues in cell surface polysaccharides^49^. This gene is in a locus similar to what has been described as *eps1* in *B. cereus* ATCC14579 coding for enzymes involved in polysaccharides biosynthesis, with a highly variable part containing different glycosyltransferases^50^.

**Fig. 5:**
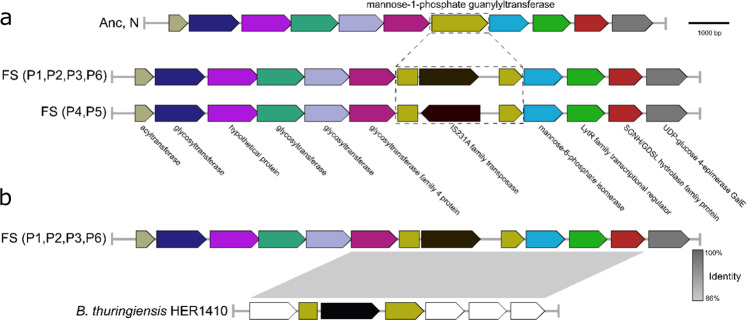
Comparative genomic characterization reveals insertion sequence plays an important role in the evolutionary adaptation of Bt407. **a** Genetic properties of the disrupted guanylyltransferase gene. **b** Identification of a similar genetic structure of the natural isolate, *B. thuringiensis* HER1410 with a guanylyltransferase disrupted by a homologous insertion sequence.

Alignment results showed that in FS morphotypes, the guanylyltransferase-encoding gene was interrupted by an insertion sequence (IS) element, annotated as IS4-like element IS231A family transposase in the genome of Bt407 (Fig. 5a). The disrupted gene is located at a genetic operon comprised of seven structural genes that are likely involved in GDP-mannose biosynthesis (Supplementary Fig. S6). Interestingly, comparing the FS isolates from the different evolved populations revealed that the orientation of IS element is reversible (Fig. 5a).

Next, to examine if such an identical insertion sequence could be detected in this gene (mannose-1-phosphate guanylyltransferase) within *Bacilli*, all complete *Bacillus* genomes are available on NCBI (785 in total) were blasted against the mutated gene, the insertion sequence, and the disrupted locus sequences. Overall, 20 genomes seemed to harbor the guanylyltransferase gene including one genome that contains an interrupted mannose-1-phosphate guanylyltransferase gene by a mobile element annotated as IS4 family transposase, which shares 74.6% similarity to the IS4-like element IS231A family transposase (Fig. 5b). The outlier genome (NZ_CP050183.1) with IS4 insertion was *B. thuringiensis* strain HER1410, a strain that has been previously used to test various bacterial phages^51^. Our in silico analysis, while preliminary due to the lack of direct experimental examination of strain HER1410, highlights the plasticity of mannose-1-phosphate guanylyltransferase and possible contribution to the adaption under certain environments.

### Mannose-1-phosphate guanylyltransferase influences the surface properties of Bt407

**Fig. 6:**
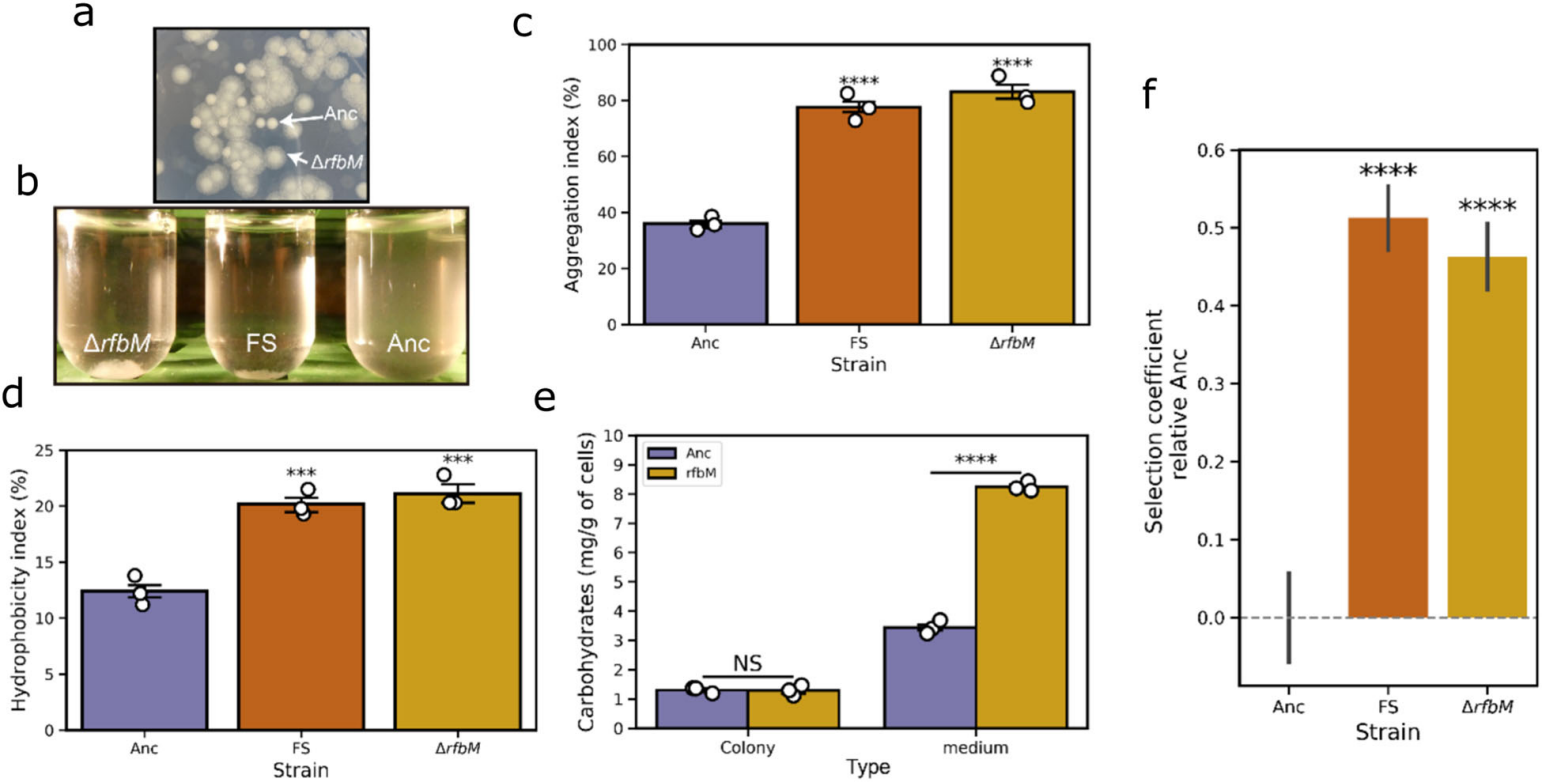
Phenotypic characterization of the mutant Bt407Δ*rfbM* strain compared with the ancestor. **a** The easily recognizable fuzzy spreader colony morphology by Bt407Δ*rfbM* suggests that disruption of the guanylyltransferase coding gene is responsible for the FS morphotype. **b** Aggregation phenotypes of Bt407Δ*rfbM* and FS variant compared with the ancestor. Aggregation (**c**) and hydrophobicity (**d**) index were characterized as described in the materials and methods (***p < 0.001, ****p < 0.0001; One-way ANOVA followed by Dunnett’s multiple comparison tests between the ancestor as control and other groups). **e** Total carbohydrates determination of Bt407Δ*rfbM* and the ancestor. Asterisks indicate significant differences between each group and the ancestor (****p < 0.0001; Student’s unpaired two-tailed *t*-test was performed between samples within each condition). Error bars indicate standard error of the mean of independent biological samples (*n* = 3). **f** Fitness of Bt407Δ*rfbM* and FS morphotype in a 1:1 competitive experiment against the ancestor. Error bars indicate the standard error of the mean (*n* = 6). Asterisks indicate a significant difference between the tested strains and the ancestor (****p < 0.0001; Student’s unpaired two-tailed *t*-test).

To elucidate whether the loss-of-function of *rfbM* is alone responsible for the observed FS morphotype, the complete gene was deleted in the ancestor Bt407 background by homologous recombination. The constructed mutant, Bt407Δ*rfbM*, demonstrated archetypal fuzzy colony morphotype on LB agar plates verifying that the loss-of-function mutation in *rfbM* could create FS phenotype (Fig. 6a). To verify whether the insertion and deletion mutations resulted in similar transcriptional patterns of the operon, quantitative real-time RT-PCR was performed for the two genes (mannose-6-phosphate isomerase as *manA* and LytR regulator as *lytR*) that located downstream of *rfbM*. As shown in Supplementary Fig. 7, quantitative real time PCR demonstrated comparable transcriptional levels of *manA* and *lytR* in samples of FS variant and Bt407Δ*rfbM* compared with control samples (ancestor). In various Gram-negative organisms, mannose-1-phosphate guanylyltransferase is involved in the biosynthesis of the capsular polysaccharide and is associated with the LPS structure^52–55^. Disruption of the gene leads to the modification of the membrane elements and cell surface properties, thus affecting cell-cell interaction and biofilm architecture. According to KEGG orthology, in *Bacilli* species, mannose-1-phosphate guanylyltransferase is responsible for mannose metabolism, glycan, and O-antigen nucleotide sugar biosynthesis, which also tightly regulate the surface properties. To experimentally examine whether *rfbM* contributes to physical cell-cell interactions, auto-aggregation properties of FS and Bt407Δ*rfbM* were quantitatively assessed. While the parental strain demonstrated very little auto-aggregation, both the Bt407Δ*rfbM* mutant and evolved FS isolate exhibited enhanced auto-aggregation (Fig. 6b, c). ImageJ analysis of the particle sizes for *rfbM* mutant and FS variant revealed a significant difference (*p* < 0.01 by Student *t*-test) in aggregate structures (38.54 mm^2^ ± 14.47 mm^2^ versus 24.48 mm^2^ ± 19.96 mm^2^ in area size, respectively). In addition to the identical morphology of the colonies, this result confirmed the phenotypic consilience among the fuzzy spreader and the *rfbM* deletion mutant.

Hydrophobicity is an important trait that is affected strongly by cell surface structures^56–58^. Throughout this study, biofilm assays were conducted using hydrophobic objects such as nylon beads and polystyrene plates, and increased biofilm formation of FS morphotypes suggested increased hydrophobicity. MATH test was performed using the parental strain, Bt407Δ*rfbM* mutant and FS isolate to quantitatively assess the ability of bacterial adhesion to hydrocarbons. As shown in Fig. 6d, the parental strain displayed a low level of hydrophobicity, with only 13% of cells partitioned into the hydrocarbon phase. On the contrary, a higher proportion of Bt407Δ*rfbM* mutant and FS isolate partitioned into the hexadecane solvent, indicating the mutation led to a cell with an increased hydrophobic cell surface.

Convergent insertion in *rfbM* of FS morphotypes across independent populations led to the hypothesis the gene is responsible for the altered phenotypes. Moreover, we questioned whether disruption of this gene is directly affected and therefore the fitness of the constructed mutant Bt407Δ*rfbM* was assayed. We tested the fitness of the mutant in a 1:1 competitive assay against the ancestor. Not surprisingly, the results indicated that Bt407Δ*rfbM* had significantly higher fitness in competition with the ancestor (*p* < 0.0001, two-tailed *t*-test), where the selection coefficient (s) of Bt407Δ*rfbM* was 0.46 per generation (Fig. 6f). Similarly, the selection coefficient of the FS variant is 0.51, as calculated by the regression model. The convergent mutations and similar increased fitness of Bt407Δ*rfbM* and FS variant suggested that the gene played a central role in adaptation to the bead system.

In summary, the enhanced multicellular trait of FS morphotype was possibly influenced by altered characteristics of the cell surface, higher level of auto-aggregation, and hydrophobicity. More specifically, the activity of the mannose-1-phosphate guanylyltransferase affects the cell surface properties and the disruption of *rfbM* by the insertion element created the observed fuzzy spreader morphotype.

## Discussion

Since the first report introducing the bead-based experiment evolution setup^25^, which primarily focused on the ecological mechanisms that sustained biofilm diversity, numerous follow-up studies have validated the robustness of this model by describing the mutational patterns^59^, genetic properties^53,60–63^, niche complementarity^64,65^, and antibiotic resistance^66,67^. Unlike other laboratory-based experimental evolution systems, this simple method is useful for modeling the complex biofilm cycle, including initial attachment, biofilm maturation, dispersal, and recolonization^68,69^.

Previously, we have investigated the evolution of *B. thuringiensis* 407 biofilms on plants by repeated selection for root-associated biofilm cycles^31^. The bead biofilm model provides a simpler, abiotic selection system to reveal how adaption to the biofilm life cycle influences bacterial evolution. Although genetic and phenotypic differentiation was already observed with the plant-adapted isolates, the bead-based model created highly predictable multicellular phenotypic differentiation. Namely, a morphologically distinct variant was observed in each population that was distinct from the ancestor, exhibiting a large colony and fuzzy appearance. Adaptive diversification has a pivotal contribution to the evolution of the bacterial community, mostly influencing biological diversity^70^. Spatially structured environments are a critical factor that facilitates such variation that allows competition of variant morphotypes and emergence of newly evolved niche-specialists, so-called ecotypes. In addition to the bead model and static microcosms that were used for Gram-negative bacteria^15,71,72^, diversification was also observed among Gram-positives, e.g., laboratory evolution of *B. subtilis* in static, shaking, and pellicle conditions^26–28^. Using a host-associated setup, Blake et al. illustrated how *B. subtilis* diversified into several morph variants when evolving in plant root-associated biofilms^29^.

The number of detected mutations was significantly less compared with our previous plant-associated experimental evolution model. This might be due to the large population size and lower selection force the bacteria were subjected to the bead system. Importantly, Spo0F acquired different mutations in most evolved isolates, suggesting sporulation is a crucial trait that influences the adaptation during the biofilm formation and dispersal cycle. This phenotypic loss of or altered sporulation can be regarded as a tradeoff between sporulation and other phenotypes related to fitness. In *B. subtilis*, evolved non-sporulating derivatives had higher growth rates than the ancestor, which demonstrated a tradeoff between sporulation and growth rates^46^. Furthermore, in addition to the insertion of the IS element, parallel mutations in FS were characterized in a cupin domain-containing protein-coding gene. These parallel IS element rearrangements and mutations in this specific gene suggest a strong fitness advantage for the mutated genotypes. Therefore, the FS morphotypes were likely acquired by the mutations in the cupin domain-containing protein, which is characterized as the mannose-1-phosphate guanylyltransferase. Interestingly, the disrupted gene is within a large locus, which was recently characterized as *eps1* in *B. cereus* ATCC 14579. The locus is highly variable due to the variety of glycosyl transferases located within this gene cluster, potentially creating an important role for these genes in the adaptation of *B. cereus* to different environments. Compared with the acquired parallel mutations in the FS morphotype, no apparent convergent mutations in the N morphotype were found. We speculated that the *N* variant is not the convergent outcome of experimental evolution, and the six selected N morphotypes are distinct genotypes. This is consistent with the different capabilities in biofilm formation of the six *N* variants. On the contrary, FS variants behaved consistently across independent populations, indicating the significant impacts brought by the convergent mutations in genes, mainly the mannose-1-phosphate guanylyltransferase.

Here, a unique Fuzzy Spreader was identified that displayed specific cellular behavior phenotypes, including altered swimming, surface spreading, biofilm formation in addition to an overall enhancement of bead colonization. Interestingly, FS variants formed dendritic or branched patterns on EPS or Trb media with 0.7% agar. In *B. cereus*, dendrite colony formation was induced by low-nutrient conditions like EPS and was connected to the production of biosurfactant compounds, which was negatively regulated by PlcR regulon in *B. cereus* ATCC 14579^73^. The extensive dendritic pattern of FS variants suggests an efficient translocation strategy, which may contribute to colonizing and surviving in a new environment. While the FS variant exhibited distinct differentiation properties compared to the ancestor and the evolved *N* variant, both FS and N morphotypes from P1 demonstrated elevated Congo red binding property, suggesting higher production of the biofilm matrix. Enhanced biofilm matrix production might promote colony expansion of the FS variants on a 0.7% agar medium. Indeed, sliding in *B. subtilis* depends on the production of both surfactant compounds as well as exopolysaccharides^74–78^. Surfactin facilitates colony expansion by reducing the friction between cells and their substrate. In *B. subtilis*, cell collectives are organized as bundles, termed as van Gogh bundles, which depend on the synergistic cooperation of surfactin-producing and matrix-producing cells^78^. Hence, whether FS variants display enhanced sliding or flagellum-dependent swarming requires further studies using directed microscopy and mutant generation. Interestingly, a trade-off between swimming motility and biofilm formation was observed in the *N* variant, other than the typical trade-off between planktonic growth and biofilm formation in the FS variant. Additionally, several independent EE models identified similar motility and biofilm trade-off^29,30^, suggesting that it is a general adaptive strategy in this selective environment, where swimming motility is a redundant trait. Our findings further support the theory that different trade-offs of evolved variants in competitive capacity drive adaptive diversification.

Intriguingly, FS variants exhibited distinct biofilm development in LB and EPS medium. The dissimilar biofilm on these two media might be caused by different amounts and compositions of nutrients, aggregation of FS variants is for example induced by nutrient-limited environments. Multicellularity confers bacteria distinct characteristics that can be advantageous or disadvantageous depending on the niche they occupy; therefore, it is interesting to determine what evolutionary factors promote multicellular groups instead of dispersed unicellular populations? Series of evolution experiments have been developed both in vivo and in silico to demonstrate how multicellularity can evolve^24,72,79–83^. For instance, multicellularity evolved 2.5 billion years ago in cyanobacteria^84^. Cyanobacteria were shaped by various environments and diversified through distinct evolutionary paths, resulting in both unicellular and multicellular forms that are found also today^85,86^. This implies that multicellularity readily evolves through adaptive diversification, microbes could diversify into certain phenotypic and genotypic variants in specific niches.

One of the emerging functions of multicellularity is the improved performance of the group compared to the undifferentiated population. Cooperative interaction could be indeed detected between the FS and N morphotypes when forming mixed biofilm compared with the biofilms created by one of evolved type or the ancestor strain alone. Further, cooperative and competitive cell–cell interactions shape the spatial assortment of genotypes within a biofilm community^40^. Our CLSM analysis of mixed biofilms revealed that FS variants colonized the beads in a segregated fashion when co-cultivated with evolved *N* variant compared with the aggregated distribution when combined with the ancestor, potentially suggesting the emergence of a synergistic interaction between the evolved morphotypes. Moreover, the high competitiveness of FS variants in the presence of the ancestor suggests increased translocation ability of FS variants, which might correlate with the increased surface spreading. Individual-based modeling has previously suggested that the ability of extracellular polymer production confers a strong competitive advantage within mixed genotype biofilms^87^.

Interestingly, morphologically similar variants to the FS type observed in this study were also identified by Rainey et al.^15^ after the evolution of *P. fluorescens* in a static microcosm allowing spatially heterogeneous diversification. It has been suggested that the fuzzy-spreader morphotype of *P. fluorescens* exhibits niche specialization by forming submerged aggregates at the bottom of tube^88–90^. Like wrinkly-spreader, fuzzy-spreader displays a multicellular phenotype including increased biofilm formation ability^72^. However, the selective advantage of the fuzzy-spreader morph was not clarified while the wrinkly-spreader was comprehensibly examined by Rainey group^36,91–93^. These studies laid a foundation for subsequent phenotypic and genotypic characterization of fuzzy-spreader^19^, demonstrating that fuzzy-spreader follows an ecological cycle of forming cellular rafts, collapsing into the bottom of the vial and reforming the rafts in the microcosms, which displays analogy to the repetitive life cycle of FS morphotype in this study. The genetic dissection of fuzzy-spreader of *P. fluorescens* identified a gene, *fuzY*, which encodes a β-glycosyltransferase that is predicted to modify O antigens of lipopolysaccharide (LPS)^19^. Mutation in *fuzY* leads to cell flocculation. Essentially, LPS defects were associated with stronger adhesion of cells both to abiotic surfaces and among each other. Similarly, the FS morphotypes of *B. thuringiensis* harbored an insertion element within a gene encoding a mannose-1-phosphate guanylyltransferase. The disrupted *rfbM* gene is in an operon that is predicted to be responsible for polysaccharides biosynthesis. In *Burkholderia* and *Pseudomonas* genera, disruption of LPS biosynthesis may improve biofilm properties such as adhesiveness, cohesiveness, and viscoelasticity^94,95^. Even though LPS has a crucial function in Gram-negatives, cells of Gram-positive bacteria are not encased by a lipopolysaccharide layer, rather having a complex layered structure including peptidoglycan, peptides, and amino acids^96,97^. Nevertheless, in Gram-positive bacteria, mannose-1-phosphate guanylyltransferase is involved in the biosynthesis of capsular polysaccharides (CPS), which are polysaccharides associated with the cell surface^98^. In *B. cereus* ATCC14579, a genomic region reported to be responsible for the synthesis of a capsular polysaccharide (*eps2*) was characterized and biofilm tests revealed that the products of the *eps2* region have a mild role in biofilm formation^50,99^. Although the locus identified in this study is not homologous to *eps2*, the variability of glycosyl transferases suggested a possible role of capsular polysaccharides in the adaptation of the bacterium to different environments.

IS element-influenced genomic rearrangements are often observed in natural isolates of *B. thuringiensis*^100–102^. Such property is considered to be a dynamic genomic state, which plays an important role in environmental adaptation and biological interactions of the species^103^. In the reference genome of Bt407, two copies of the IS4-like element gene are present, revealing the possible plasticity of the genome and IS element’s contribution to adaptability. Mobile elements pose two mechanisms of movement, conservative (non-replicative) and replicative pathways. Members of the IS4 family contain a conserved transposase-integrase motif, among which IS231A demonstrates a non-replicative mode of transposition exclusively^104^.

Cellular hydrophobicity and auto-aggregation are highly dependent upon non-polar functional elements, surface protein, pilus, and capsules. It has been previously emphasized that CPS plays an essential role in determining biofilm size by various mechanisms such as inhibiting continual growth of matured biofilms via quorum sensing^57,105,106^. Variation of such membrane-associated structure has been shown to directly influence bacterial surface properties, thus cell–cell and cell–surface interactions that influence biofilm architecture in numerous bacteria^107,108^. In *Porphyromonas gingivalis*, a mutation in a glycosyltransferase gene was responsible for enhanced biofilm formation and hydrophobicity because of the adjustment of cell surface characteristics^109^.

In summary, our work provides the first comprehensive assessment of the evolutionary diversification of *B. cereus* associated with abiotic biofilms. The findings reported here shed new light on the previously unknown adaptive strategy of *B. cereus* by diversifying into phenotypically distinct morphotypes in response to biofilm life cycles. FS variant exhibited distinct multicellular phenotypes with specifically increased biofilm development. FS variant retained the highest fitness and acted as a generalist that balances biofilm formation and dispersal due to a disrupted guanylyltransferase-coding gene. Genomic characterization of FS morphotypes revealed parallel genomic plasticity via displacement of an insertion element that promoted evolutionary adaptation. Multicellular-like behavior of generalists during selection within a biofilm lifecycle could be a relevant evolutionary adaptation in environmental niches such as plants and soil, in which the biofilm lifecycle is suggested to be required for the survival of bacteria.

## Methods

### Bacterial strains and growth conditions

The ancestral (wide-type) strain used in this study is *B. thuringiensis* 407 (Bt407 Cry-, commonly referred to as Bt407). Table 1 includes all bacterial strains, plasmids, and primers used in this study. *Escherichia coli* XL1-Blue was used for molecular cloning experiments. Routinely, bacterial strains including *B. thuringiensis* and *E. coli* were cultured in lysogeny broth (LB-Lennox, Carl Roth; 10 g/L tryptone, 5 g/L yeast extract, and 5 g/L NaCl) plates solidified with 1.5% agar or stored at −80 °C with 28% glycerol added to an overnight culture. When required, concentrations of antibiotics were used as indicated: kanamycin (50 µg/mL), ampicillin (100 µg/mL), erythromycin (5 µg/mL), and tetracycline (10 µg/mL). X-gal (5-bromo-4-chloro-3-indolyl-β-d-galactopyranoside) was used at 40 µg/mL. For all relevant assays, the incubated cultures were vortexed vigorously to ensure proper disruption of any cellular aggregates.

**Table 1:** Information of strains, plasmids, and oligos used in this study.

	Description	Reference
*Strains*		
*B. thuringiensis* 407 Cry- (Bt407)	Acrystalliferous *B. thuringiensis* type strain	^111^
Bt407 mKate	Bt407 transformed with pTB604	^31^
Bt407 FS GFP	Bt407 evolved FS variant from P1 transformed with pTB603	This study
Bt407 N mKate	Bt407 evolved N variant from P1 transformed with pTB604	This study
Bt407*ΔrfbM*	Bt407 introduced a deletion mutation of *rfbM*	This study
*E. coli* XL-1 Blue	*recA1 endA1 gyrA96 thi-1 hsdR17 supE44 relA1 lac* [F ´ *proAB lacI*q*Z*∆*M15* Tn*10* (Tet^R^)]	^129^
*Plasmids*		
pMAD	Shuttle vector, *bgaB*, *bla*, *ermC* (Amp^R^ and Ery^R^)	^115^
pMAD (I-SceI)	pMAD containing I-*Sce*I digestion site (Amp^R^ and Ery^R^)	^116^
pBKJ223	A vector expressing the I-*Sce*I enzyme (Amp^R^ and Tet^R^)	^114^
pTB603	pNW33N with P_hyspank_-GFP	^31^
pTB604	pNW33N with P_hyspank_-mKATE2	^31^
*Oligos*		
oYL51	Forward oligo to check if a mutation is present in BTB_RS26870 CCGGAATTCTCTAGCTACTCTCACTACT	This study
oYL52	Reverse oligo to check if a mutation is present in BTB_RS26870 CGCGGATCCCTAGGGTGAGGAGATAATA	This study
oYL55	Forward oligo to amplify left arm of the homologous fragment with EcoRI site CCG GAA TTCAACCTTATAATCCTCATGGG	This study
oYL56	Reverse oligo to amplify left arm of the homologous fragment with XbaI site GCTCTAGAGCAATGGAAAGAAATAGAGG	This study
oYL57	Forward oligo to amplify right arm of the homologous fragment with XbaI site GCTCTAGATATTATCTCCTCACCCTAGA	This study
oYL58	Reverse oligo to amplify right arm of the homologous fragment with BamHI site CGCGGATCCTTATGCTTGGGATTTGCAAC	This study
oYL49	Forward oligo for sequencing the insert of multi cloning sites of pMAD TCTATCGATGCATGCCAT	This study
oYL50	Reverse oligo for sequencing the insert of multi cloning sites of pMAD AGAATCATAATGGGGAAGG	This study
qBCE3	Forward oligo to amplify the housekeeping gene *rpoA* CGTGGATATGGTACTACTTTGG	^125^
qBCE4	Reverse oligo to amplify the housekeeping gene *rpoA* TTCTACTACGCCCTCAACTG	^125^
oYL61	Forward oligo to amplify the housekeeping gene *udp* ACTAGAGAAACTTGGAAATGATCG	^130^
oYL62	Reverse oligo to amplify the housekeeping gene *udp* GACGCTTAATTGCACGGAAC	^130^
oYL63	Forward oligo to amplify the gene *manA* for RT-qPCR CTTACGGTTCTTAATGTCGC	This study
oYL64	Reverse oligo to amplify the gene *manA* for RT-qPCR CGTATTGATAGTGATGGGAA	This study
oYL65	Forward oligo to amplify the gene *lytR* for RT-qPCR TAGGAGTGCTGATTATTGGT	This study
oYL66	Reverse oligo to amplify the gene *lytR* for RT-qPCR AGAATCTGATCGTCCTACC	This study

To determine the growth parameters, strains were firstly recovered in LB medium and incubated overnight at 37 °C. The bacterial cultures were centrifuged, and pellets were washed, then resuspended in an EPS medium. One milliliter of density-adjusted bacterial cultures (optical density, OD ~0.05) was transferred into a 24-well plate allowing limited oxygen exchange. The plate was incubated at 30 °C with continuous shaking at 90 rpm in a plate reader (BioTek Synergy HTX Multi-Mode Microplate Reader), and OD reads at 600 mm were captured every 10 min.

### Experimental evolution

Experimental evolution setup was applied as previously described with modifications^25^. A homogenous colony of Bt407 ancestor was inoculated into 24-well microtiter plates containing a nylon bead floating in EPS medium^110^, which was low-nutrient medium specifically for *B. cereus* biofilm formation (7 g/L K_2_HPO_4_, 3 g/L KH_2_PO_4_, 0.1 g/L MgSO_4_·7H_2_O, 0.1 g/L (NH4)_2_SO_4_, 0.01 g/L CaCl_2_, 0.001 g/L FeSO_4_, 0.1 g/L NaCl, 1 g/L glucose, and 125 mg/L yeast extract). The plates were sealed with parafilm to prevent moisture loss and allow gas exchange and then incubated at 30 °C with continuous shaking at 90 rpm. After 24 h, the colonized bead was transferred into a fresh EPS medium containing two uncolonized, sterile beads. To distinguish the new beads and the old ones, two sterile marked or unmarked beads were used for each transfer. Around every three transfers, biofilm was dispersed from one of the newly colonized beads using rigorous vortexing and subjected to standard biofilm productivity analysis that is based on determining the total cell number. The whole experiment included six individually evolved populations and additional six planktonic bacterial cultures as a control without beads. Serial transfers of control populations were performed via 1:100 dilutions of planktonic cultures incubated in EPS medium for 24 h.

### Genetic manipulations

Electroporation of *B. thuringiensis* and DNA extraction were performed as standard procedures^111,112^. Restriction enzymes, T4 DNA ligase, and Phusion High-Fidelity DNA Polymerase were purchased from Thermo Scientific. DNA fragments were purified by using NucleoSpin Gel and PCR Clean-up kits (Macherey-Nagel). Oligos were synthesized by TAG Copenhagen A/S and DNA sequences were sequenced at Eurofins Genomics. For *E. coli*, standard molecular was applied according to standard protocols^113^.

The deletion mutant of *B. thuringiensis* was constructed by homologous combinations using a mark-less replacement method introduced by Janes and colleagues^114^. Briefly, the flanking homologous fragments of approximately 700 bp were amplified and cloned into the shuttle vector pMAD^115^ with an additional I-SceI restriction site^116^. The plasmid carrying the flanking homologous fragments was electroporated into Bt407 to obtain blue transformants on LB agar plates containing erythromycin and X-gal. Facilitation of the integration into the chromosome was conducted by shifting incubation of the culture to a replication-nonpermissive temperature^114^. Subsequently, pBKJ223, which encodes I-SceI restriction enzyme, was transformed to induce a double-stranded break at the chromosome thus promoting a second recombination event^117^. Finally, White colonies represented as those that have lost erythromycin resistance were selected and genomic DNA was extracted. The mutation was verified by PCR and Sanger sequencing.

### Fitness assays of bead biofilms

Competitive fitness assays in bead biofilms were determined between the evolved variants (FS and N) and the ancestor where the ratio of Malthusian parameters (*m*) was calculated as described by Lenski et al.^38^. Briefly, a mixture of overnight LB cultures (OD = 0.02) of evolved variants and the ancestor (1:1 ratio) was used to inoculate the initial EPS medium. After one or two cycles of colonization, bacteria were recovered and plated onto agar plates and calculated as

1 $$m = \ln (N_1/N_0),$$

where *N*_1_ and *N*_0_ represented colony forming units counted in the end and at the start of the assays, respectively. To differentiate the strains, FS and N variants were transformed with GFP and mKate fluorescent reporters, respectively. Relatively, ancestors that carry GFP and mKate fluorescent reporters were used.

The selection coefficient was calculated according to the regression model^71^:

2 $${{{{s}}}} = \left[ {{{{\mathrm{ln}}}}\left( {{{{{R}}}}\left( {{{\mathrm{t}}}} \right)/{{{{R}}}}\left( 0 \right)} \right)} \right]/{{{\mathrm{generation}}}},$$

in which *R* is the ratio of competition strains against the ancestor. Generation time was estimated according to

3 $${{{\mathrm{ln}}}}\left( {{{{{P}}}}_{{{\mathrm{f}}}}/{{{{P}}}}_{{{\mathrm{i}}}}} \right)/{{{\mathrm{ln}}}}\left( 2 \right),$$

where *P*_f_ and *P*_i_ represent the final and the initial population, respectively.

### Phenotypic characterization of genetic variants

Congo red indicator assay: 10 µL overnight grown bacterial culture was spotted onto LB agar plate (1.5%) supplemented with 40 µg/mL of Congo red (CR) and 20 µg/mL of Coomassie brilliant blue dyes. Bacterial colonies were grown at 30 °C, after which images were taken using Panasonic DC-TZ90 camera. Colonies were resuspended in LB and OD_600_ was used to access the cell density. For CR bound, cells were resuspended in one milliliter of 0.005% (w/v) CR and incubated for 2 h at 30 °C. Samples were centrifuged and OD_490_ value of the supernatant was determined and compared with appropriate CR standards to obtain mg CR^118^.

Surface motility assay: 10 µL overnight culture (approx. 10^6^ CFU) was spotted onto the center of 0.7% agar plate containing a nutrient medium and incubated at 30 °C for up to 72 h. Colony images were recorded with a Panasonic DC-TZ90 camera. EPS agar plates were made with EPS medium supplemented with agar (7.0 g/L K_2_HPO_4_, 3.0 g/L KH_2_PO_4_, 0.1 g/L MgSO_4_·7H_2_O, 0.1 g/L (NH_4_)_2_SO_4_, 0.01 g/L CaCl_2_, 0.001 g/L FeSO_4_, 0.1 g/L NaCl, 1.0 g/L glucose, and 125 mg/L yeast extract)^73^. TrB medium contained 1% tryptone and 0.5% NaCl^119^. The swimming ability of different strains was tested on LB agar plates (0.3%). Similarly, 10 μL culture with approximately 10^6^ cells was spotted onto the center of the plates and incubated at 30 °C for 12 h. Each experiment was performed in triplicate.

For measurement of the aggregate sizes, ImageJ-Fiji was applied to images in triplicates using circular cell sections to avoid tilted selection.

Biofilm visualization: Bacterial strains were inoculated in rich LB medium LB and incubated for 24 h at 30 °C. Biofilms forming on the tube walls were imaged with a Panasonic DC-TZ90 camera.

Biofilm dispersal and CV staining: Dispersal was quantified according to previous report^45^. Bacterial cultures were incubated in EPS medium for 24 h to form mature biofilms in 24-well microtiter plates, followed by a gentle wash of the unattached cells. One milliliter of fresh EPS medium was added into the plates, which was then agitated vigorously for 1 and 10 h represented as weak and strong disturbance, respectively. The remaining biofilms were stained by crystal violet (1%) and solubilized by ethanol, after which A490 was documented.

### Microscopy

For bright-field images of bacterial pellicles and colonies, Axio Zoom V16 stereomicroscope (Carl Zeiss) was used, which was equipped with a Zeiss CL 9000 LED light source, a PlanApo Z 0.5× objective, and AxioCam 591 MRm monochrome camera (Carl Zeiss, Jena).

Confocal laser scanning microscopy (CLSM) was conducted using a Leica Microsystems Confocal Microscope SP8. For surface-attached biofilms imaging, bacterial cultures were incubated in high content imaging plate (Corning, New York) to form mature biofilms as described above. Afterward, biofilms were washed with sterilized ddH2O twice to remove non-attached aggregates. CLSM images were obtained using a 63×/1.4 OIL objective. Fluorescent excitation was conducted with the argon laser at 488 nm and the emitted fluorescence was acquired at 484–536 and 567–654 nm for GFP and mKate, respectively. *Z* stack series of biofilms were obtained with 1 μm steps and stacked images were merged using ImageJ software. For frequencies determination of strains in each CLSM image, ImageJ was used to convert the stacks into binary images and the threshold was set above 0. Afterward, the generated stacks were activated using the biofilm selection and the total pixel volumes for each stack were retrieved using the “stacks statistics” function^120^.

### Whole-genome sequencing and hybrid assembly

A 4 mL aliquot of bacterial cultures of 12 evolved isolates (representative FS and *N* variants from six evolved populations) plus the ancestral strain was centrifuged and genomic DNA was extracted using GeneMATRIX Bacterial Genomic DNA Purification Kit (EURx Ltd, Poland). Paired-end libraries were prepared using the NEBNext® Ultra™ II DNA Library Prep Kit for Illumina sequencing. Illumina NextSeq sequencer was used to generate paired-end fragment reads using TG NextSeq® 500/550 High Output Kit v2 (300 cycles).

Long-read libraries were constructed using Rapid Barcoding Sequencing kit (SQK-RBK004) and long-reads were generated on MinION Mk1B (Oxford Nanopore Technologies). Raw sequences were base called using MinKNOW sequencing software (Oxford Nanopore Technologies). Preprocessing was performed using AdapterRemoval (v2.3.1) and Filtlong (v0.2.1) for short and long reads, respectively.

Subsequently, reads of each isolate were hybrid assembled with short reads from Illumina sequences by using Unicycler (v0.4.9)^121^ with default settings. Assembled genomes of evolved isolates and the ancestor were aligned using MAFFT (v7.4)^122^ with the auto option (mafft --auto input > output). Finally, assemblies were visualized in the CLC workbench (v 9.5.1) and annotated by Prokka (v1.14.5) using Bt407 as the reference genome. Easyfig (v2.1) was used for visualization of the genomic alignment^123^.

As a reference, the assembled genomes were compared to *B. thuringiensis* 407 genome (GenBank accession no. CP003889.1), after extraction of previously mapped SNPs found in the ancestor used in this study. SNPs were identified between the re-assembled ancestral genome and the assembled contigs using Snippy (GitHub - tseemann/snippy at v4.6.0) with default settings. Identified mutations are included in Supplementary Table 1. Raw sequencing data have been deposited to the NCBI Sequence Read Archive (SRA) database under BioProject accession number: PRJNA757963.

Profiling of *Bacillus* genomes for disrupted *rfbM* genes was done by downloading all completed *Bacillus* genomes from NCBI using ncbi-genome-download (https://github.com/kblin/ncbi-genome-download) and searching for the intact gene, the IS, and the interrupted gene using blastn.

### Quantitative RT-PCR

For RNA extraction^31^, overnight cultures were diluted to an OD_600_ of 1 in LB medium and cultivated to late log phase. Cells were collected and total RNA was purified by using a phenol–chloroform–isopropanol method with a High Pure RNA isolation kit (Roche, Germany)^124^. The quality of purified RNA was verified using Nanodrop and gel electrophoresis, which was followed by the treatment of DNase I (RNase-free, Fermentas) in a buffer for 1 h at 37 °C. Reverse transcription was performed to obtain cDNA with RevertAid H Minus Reverse Transcriptase (Thermo Scientific) and 50 pmol random hexamers (Thermo Scientific) on 1 μg of total RNA in duplicate for each sample^125^. For all samples, negative controls were performed in reactions without the reverse transcriptase. RT-qPCR experiments were conducted on an Mx3000P QPCR System (Agilent) in a 96-well microtiter plate with a reaction volume of 20 µL containing 10 µL of Luna^®^ Universal qPCR Master Mix (NEB), 4 µL of primers (included in Table 1), and 1 µL of cDNA. PCR reactions were performed in triplicates for each sample. The stably expressed gene, *udp* was chosen as a reference gene that was included for each sample and on each plate. The primers used are listed in Table 1. The lack of DNA contamination in RNA samples was verified by amplifying all negative control samples. The quantity of *manA* and *lytR* cDNA was normalized to the level of *udp* using the 2^−ΔΔCt^ method.

### Cell surface characterization

For adhesion to hydrocarbons (BATH) assay^56^, cells were washed and resuspended in PBS buffer to remove culturing medium. The bacterial suspension was then adjusted to an optical density (600 nm) of 0.25 ± 0.05 (recorded as *A*_0_), representing the standard number of bacteria (10^7^–10^8^ CFU/mL). Then, an equal volume of Hexadecane (Sigma-Aldrich) was added. The two-phase liquid system was mixed completely by vortexing vigorously for 10 min, followed by a 1 h of incubation at room temperature allowing stratification. The optical density (600 nm) of the aqueous phase was measured as *A*. The hydrophobicity, represented as adhesion to hydrocarbons, was calculated across three different replicates according to the formula:

4 $$\frac{{(A_0 - A)}}{{A_0}} \times 100{{{\mathrm{\% }}}}{{{\mathrm{.}}}}$$

For auto-aggregation analysis, liquid cultures were monitored^126^. Briefly, glass tubes containing 5 mL of EPS were inoculated with 24-h-old colonies and cultured overnight in a 37 °C incubator and shaking at 220 rpm. Bacterial cultures were then settled for 10 h at 4 °C. Auto-aggregation was quantified by measuring the cell density changes before and after settlement as follows:

5 $$\left( {1 - \frac{{{\mathrm{OD}}_{{\mathrm{final}}}}}{{{\mathrm{OD}}_{{\mathrm{initial}}}}}} \right) \times 100{{{\mathrm{\% }}}}{{{\mathrm{.}}}}$$

The auto-aggregation index was calculated from triplicates.

### Carbohydrates quantification

Collection of exopolysaccharide was applied according to a previous work^127^. Briefly, strains were cultivated at 30 °C for 48 h on EPS agar (1.5%) plates, or in EPS liquid medium with shaking at 220 rpm. Bacterial colonies were suspended in 1 mL of 0.9% NaCl buffer and subjected to vigorous sonication (5 × 12 pulses of 1 s with 50% amplitude; Ultrasonic Processor VCX-130, Vibra-Cell, Sonics, Newtown). Liquid bacterial cultures were treated with the same sonication process. Bacterial biomass was separated by centrifugation (10 min at 12,000 × *g*) and the supernatant was collected. The exopolysaccharide content of samples was quantified by using the phenol-sulfuric acid method^128^. Standard curve was constructed using diluted glucose solution (*y* = 19.773*x* + 0.0827, *R*² = 0.9984).

### Statistical analysis

Unless indicated otherwise, all experiments were performed with at least three biological replicates. Statistical analysis of bacterial traits comparison between evolved isolates and the ancestor was analyzed and illustrated using Python 3.8 with Statsmodels packages or Graphpad 8. To test statistically significant differences between the means of three or more independent groups, one-way ANOVA analysis was carried out, followed by Dunnett’s post-hoc analysis. For comparing the means of two groups, students’ unpaired two-tailed *t*-test was performed.

### Reporting summary

Further information on research design is available in the Nature Research Reporting Summary linked to this article.

## Supplementary information

Supplementary Information

Reporting Summary

Supplementary Dataset 1

Supplementary Dataset 2

## Data Availability

Raw sequencing data have been deposited to the NCBI Sequence Read Archive (SRA) database under BioProject accession number: PRJNA757963. All other data that were used to create the figures and support the findings of this study are available from the corresponding author upon request.
