# Supplementary material for: Adaptation and phenotypic diversification of Bacillus thuringiensis biofilm are accompanied by fuzzy spreader morphotypes
Source: NPJ Biofilms Microbiomes. 2022 Apr 13;8:27. doi: 10.1038/s41522-022-00292-1 (PMC9007996; doi:10.1038/s41522-022-00292-1)
Supplement: Supplementary file 1 — Supplementary Information [file 41522_2022_292_MOESM1_ESM.pdf]

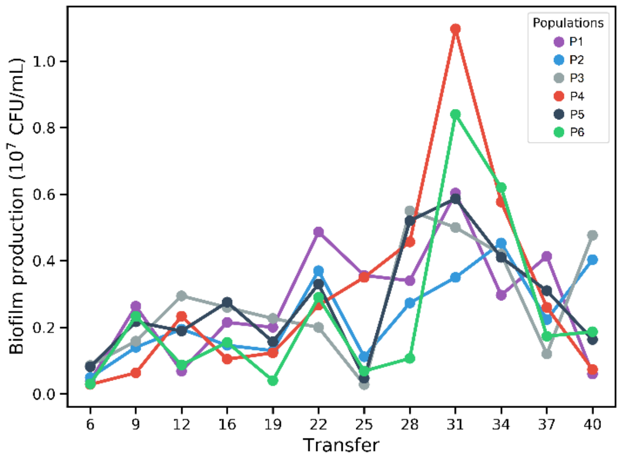

Supplementary Figure 1. Biofilm productivity of bead-colonized Bt407 (Cry$^-$) of all six evolved populations are shown at roughly every 3th transfers.

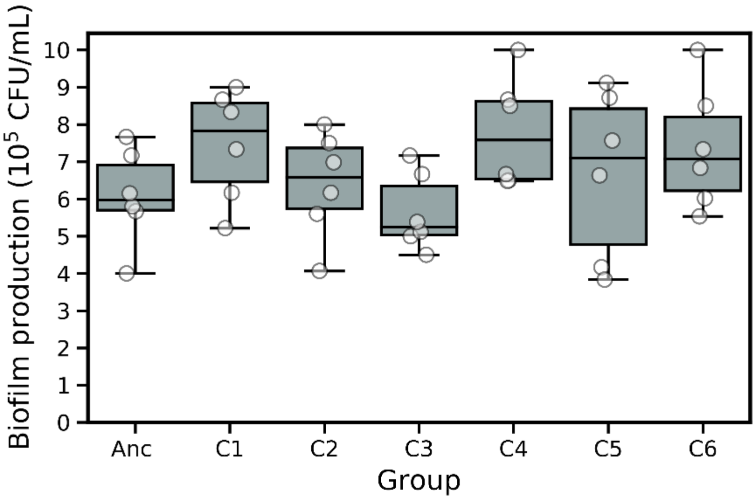

Supplementary Figure 2. Biofilm production ability of six control populations compared with the ancestor. Boxes indicate Q1–Q3, lines indicate the median, and bars span from max to min (n = 6 biologically independent samples).

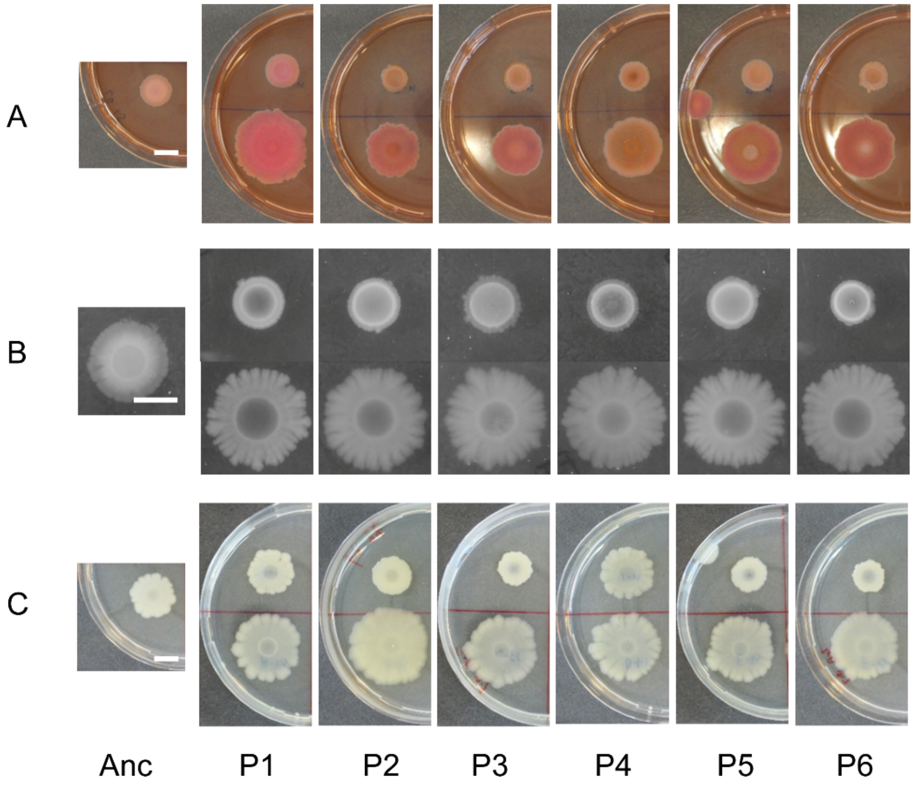

Supplementary Figure 3. The tested phenotypical characteristics of evolved morph variants from all six evolved populations are presented compared with the ancestor, including Congo red uptake colony morphologies (A), swarming motility on EPS (B) and TrB (C) media contain 0.7% agar. Top and bottom images represent N and FS evolved variants, respectively. Scale bars indicate 10 mm for all panels.

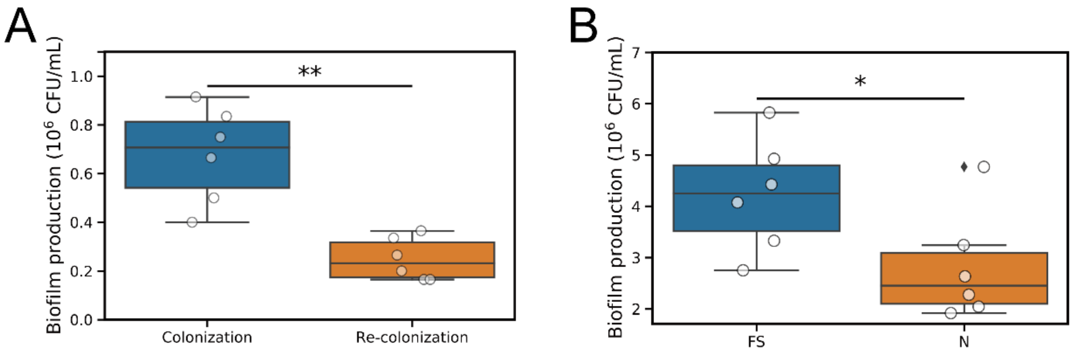

Supplementary Figure 4. (A) Statistical different biofilm productions of colonization and re-colonization in the ancestor strain. Boxes indicate Q1–Q3, lines indicate the median, and bars span from max to min (n = 6 biologically independent samples). (B) Statistically different biofilm productions of FS and N morphotypes when cultured together. Boxes indicate Q1–Q3, lines indicate the median, and bars span from max to min (n = 6 biologically independent samples). Asterisks indicate significant differences between each group and the ancestor (**p* <0.05, ***p* <0.01; two tailed t-test with Welch's corrections)

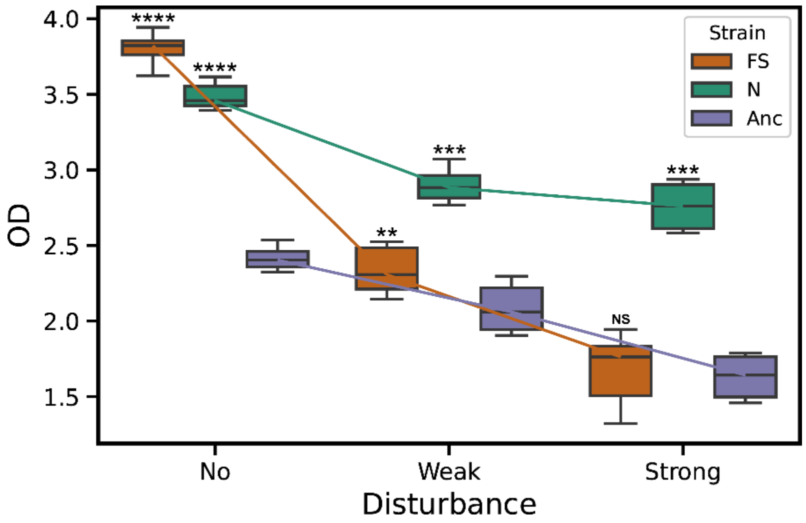

Supplementary Figure 5. Bacterial dispersal ability was quantified for the FS and N morph variants along with the ancestor (n=10). Bacterial cultures were incubated in 24-well microtiter plates. OD, an indicator of biofilm formation in the wells by crystal violet staining was monitored for three different treatment groups. Plates were vigorously shaked on an orbital shaker (220 rpm) for 1 h and 10 h, represented as weak and strong disturbance to induce biofilm dispersal, followed by staining of the biofilms with crystal violet. Asterisks indicate significant differences between each group and the ancestor (***p* <0.01, ****p* <0.001, *****p* <0.0001, NS indicates non-significant; two tailed t-test with Welch's corrections)

A

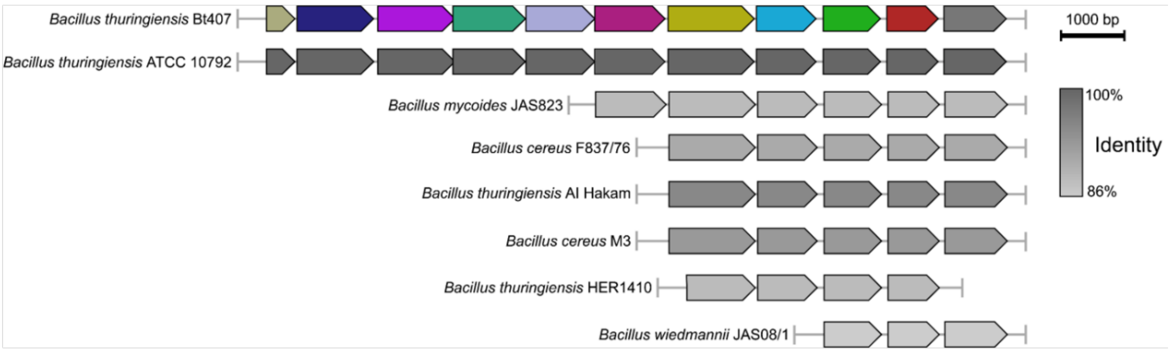

B

Operon	IdGene	Type	COGgene	PosLeft	postRight	Strand	Function
1							
	MNDAOHKI_00011	CDS	COG1316	12656	13570	-	[K] Transcriptional regulator
	MNDAOHKI_00012	CDS	COG1482	13682	14656	-	[G] Phosphomannose isomerase
	MNDAOHKI_00013	CDS	COG0836	14666	16039	-	[M] Mannose-1-phosphate guanylyltransferase
	MNDAOHKI_00014	CDS	COG0438	16087	17211	-	[M] Glycosyltransferase
	MNDAOHKI_00015	CDS	COG0438	17208	18302	-	[M] Glycosyltransferase
	MNDAOHKI_00016	CDS	COG0438	18313	19464	-	[M] Glycosyltransferase
	MNDAOHKI_00017	CDS	COG3872	19436	20665	-	[R] Predicted metal-dependent enzyme
2							
	MNDAOHKI_00018	CDS	COG0438	20730	21947	-	[M] Glycosyltransferase
	MNDAOHKI_00019	CDS	COG0110	21984	22439	-	[R] Acetyltransferase (isoleucine patch superfamily)
	MNDAOHKI_00020	CDS	COG2244	22761	24044	-	[R] Membrane protein involved in the export
	MNDAOHKI_00021	CDS	COG0677	24255	25511	-	[M] UDP-N-acetyl-D-mannosaminuronate dehydrogenase
	MNDAOHKI_00022	CDS	COG2148	25531	26211	-	[M] Sugar transferases involved in lipopolysaccharide synthesis
	MNDAOHKI_00023	CDS	COG1210	26230	27111	-	[M] UDP-glucose pyrophosphorylase

Supplementary Figure 6. (A) Comparative genomic characterization of the disrupted genetic region of Bt407 compared with the homologous regions of other species in *B. cereus* group. (B) The predicted functions in the operon are indicated, where the the disrupted gene is highlighted with a red rectangle.

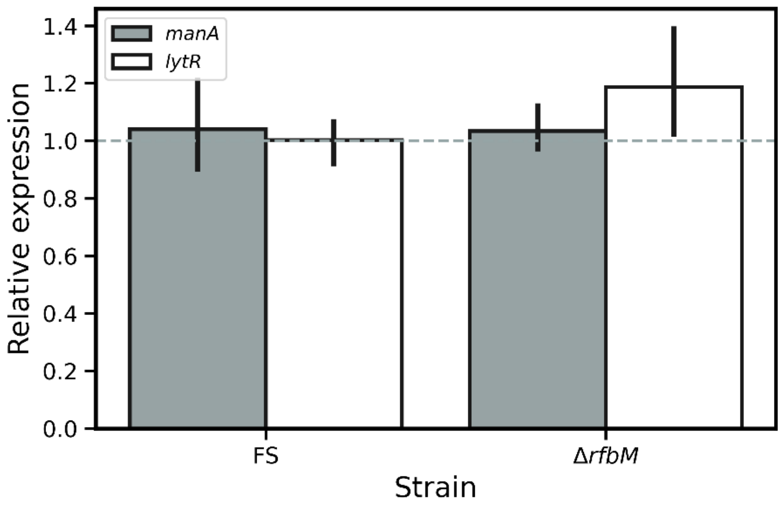

Supplementary Figure 7. RT-qPCR determination of the relative gene expression levels of *manA* and *lytR* genes. Relative expressions of the two downstream genes of *rfbM*, *manA* (gray bars) and *lytR* (white bars), were detected in bacterial cultures of Fuzzy spreader variant (FS in the figure) and Bt407*ΔrfbM* (*ΔrfbM* in the figure). Transcription levels of genes were normalized to those of housekeeping gene *udp* and compared to the transcript levels of the ancestor, which is shown as the dashed line in the figure.

Supplementary Table 1. Congo Red (CR) bound by Bt407 ancestor, the evolved isolates and recreated mutant.

Strain	CR bound (mg CR/OD_{600})	Relative CR bound
Bt407 ancestor	0.075 ± 0.021[a]	1
Evolved FS variant	0.214 ± 0.039[b]	2.8
Evolved N variant	0.091 ± 0.022[a]	1.21
Bt407*ΔrfbM*	0.196 ± 0.017[b]	2.61

Colonies grown on LB agar plates for 48h before assay. The total amount of Congo Red (CR) bound was measured by dividing bound-CR (mg) by OD_{600} of strains (mean ± SE, n = 6). One-way ANOVA analysis was carried out to examine statistical difference among strains, followed by Dunnett's post-hoc analysis. Relative CR bound is calculated relative to Bt407 ancestor.
