# Supplementary material for: Adaptation and phenotypic diversification of Bacillus thuringiensis biofilm are accompanied by fuzzy spreader morphotypes
Source: NPJ Biofilms Microbiomes. 2022 Apr 13;8:27. doi: 10.1038/s41522-022-00292-1 (PMC9007996; doi:10.1038/s41522-022-00292-1)
Supplement: Supplementary file 2 — Reporting Summary [file 41522_2022_292_MOESM2_ESM.pdf]

nature portfolio

Corresponding author(s): Akos T Kovacs

Last updated by author(s): 2022 01 04

Reporting Summary

Nature Portfolio wishes to improve the reproducibility of the work that we publish. This form provides structure for consistency and transparency in reporting. For further information on Nature Portfolio policies, see our Editorial Policies and the Editorial Policy Checklist.

Statistics

For all statistical analyses, confirm that the following items are present in the figure legend, table legend, main text, or Methods section.

n/a	Confirmed	
☐	☒	The exact sample size (*n*) for each experimental group/condition, given as a discrete number and unit of measurement
☐	☒	A statement on whether measurements were taken from distinct samples or whether the same sample was measured repeatedly
☐	☒	The statistical test(s) used AND whether they are one- or two-sided<br>*Only common tests should be described solely by name; describe more complex techniques in the Methods section.*
☐	☒	A description of all covariates tested
☐	☒	A description of any assumptions or corrections, such as tests of normality and adjustment for multiple comparisons
☐	☒	A full description of the statistical parameters including central tendency (e.g. means) or other basic estimates (e.g. regression coefficient) AND variation (e.g. standard deviation) or associated estimates of uncertainty (e.g. confidence intervals)
☐	☒	For null hypothesis testing, the test statistic (e.g. *F*, *t*, *r*) with confidence intervals, effect sizes, degrees of freedom and *P* value noted<br>*Give P values as exact values whenever suitable.*
☒	☐	For Bayesian analysis, information on the choice of priors and Markov chain Monte Carlo settings
☒	☐	For hierarchical and complex designs, identification of the appropriate level for tests and full reporting of outcomes
☒	☐	Estimates of effect sizes (e.g. Cohen's *d*, Pearson's *r*), indicating how they were calculated

Our web collection on statistics for biologists contains articles on many of the points above.

Software and code

Policy information about availability of computer code

Data collection	Paired-end libraries were prepared using the NEBNext® Ultra™ II DNA Library Prep Kit for Illumina sequencing. Illumina NextSeq sequencer was used to generate paired-end fragment reads using TG NextSeq® 500/550 High Output Kit v2 (300 cycles).<br>Long-read libraries were constructed using Rapid Barcoding Sequencing kit (SQK-RBK004) and long-reads were generated on MinION Mk1B (Oxford Nanopore Technologies). Raw sequences were base called using MinKNOW sequencing software (Oxford Nanopore Technologies).<br>Preprocessing was performed using AdapterRemoval and Filtlong for short and long reads, respectively.
Data analysis	For Illumina reads, primary data analysis (basecalling) was carried out with “bcl2fastq” software (v2.17.1.14; Illumina). All further analysis steps were done in CLC Genomics Workbench Tool 9.5.1. Reads were quality trimmed using an error probability of 0.05 (Q13) as the threshold. In addition, the first 10 bases of each read were removed.<br>For Nanopore sequenced reads, the basecalling was conducted in EPI2ME (Oxford Nanopore Technologies). Hybrid assembly was performed with Unicycler v0.4.9. Alignment was performed using Snippy v4.6.0.<br>For statistical analysis, GraphPad Prism 8 was used.

For manuscripts utilizing custom algorithms or software that are central to the research but not yet described in published literature, software must be made available to editors and reviewers. We strongly encourage code deposition in a community repository (e.g. GitHub). See the Nature Portfolio guidelines for submitting code & software for further information.

Data

Policy information about availability of data

All manuscripts must include a data availability statement. This statement should provide the following information, where applicable:

- Accession codes, unique identifiers, or web links for publicly available datasets
- A description of any restrictions on data availability
- For clinical datasets or third party data, please ensure that the statement adheres to our policy

Raw sequencing data has been deposited to the NCBI Sequence Read Archive (SRA) database under BioProject accession number: PRJNA757963. All other data that were used to create the figures and support the findings of this study are available from the corresponding author upon request.

Field-specific reporting

Please select the one below that is the best fit for your research. If you are not sure, read the appropriate sections before making your selection.

☒ Life sciences ☐ Behavioural & social sciences ☐ Ecological, evolutionary & environmental sciences

For a reference copy of the document with all sections, see nature.com/documents/nr-reporting-summary-flat.pdf

Life sciences study design

All studies must disclose on these points even when the disclosure is negative.

Sample size	No sample-size calculation was performed.
Data exclusions	No data exclusions
Replication	For precise and robust results, six independent biofilm populations and six independent planktonic populations were regarded as replicates; At the end of the experiments, six isolates with similar morphotypes were considered as replicates to represent the corresponding morphotype described later.
Randomization	Not relevant to the study
Blinding	Not relevant to the study

Reporting for specific materials, systems and methods

We require information from authors about some types of materials, experimental systems and methods used in many studies. Here, indicate whether each material, system or method listed is relevant to your study. If you are not sure if a list item applies to your research, read the appropriate section before selecting a response.

Materials & experimental systems

n/a	Involved in the study	
☒	☐	Antibodies
☒	☐	Eukaryotic cell lines
☒	☐	Palaeontology and archaeology
☒	☐	Animals and other organisms
☒	☐	Human research participants
☒	☐	Clinical data
☒	☐	Dual use research of concern

Methods

n/a	Involved in the study	
☒	☐	ChIP-seq
☒	☐	Flow cytometry
☒	☐	MRI-based neuroimaging
